# The Effect of Differentiation Induction on FAK and Src Activity in Live HMSCs Visualized by FRET

**DOI:** 10.1371/journal.pone.0072233

**Published:** 2013-08-27

**Authors:** Xiaoling Liao, Shaoying Lu, Yiqian Wu, Wenfeng Xu, Yue Zhuo, Qin Peng, Bo Li, Ling Zhang, Yingxiao Wang

**Affiliations:** 1 Biomaterials and Live Cell Imaging Institute, Chongqing University of Science and technology, Chongqing, People's Republic of China; 2 Department of Bioengineering, Institute for Genomic Biology, University of Illinois, Urbana-Champaign, Urbana, Illinois, United States of America; 3 Beckman Institute for Advanced Science and Technology, Center for Biophysics and Computational Biology, Department of Integrative and Molecular Physiology, Institute for Genomic Biology, University of Illinois, Urbana-Champaign, Urbana, Illinois, United States of America; 4 Biomedical Engineering Programme, Department of Electronic Engineering, Chinese University of Hong Kong, Shatin, NT, Hong Kong, People's Republic of China; 5 Department of Bioengineering, University of California San Diego, San Diego, California, United States of America; Georgia Regents University, United States of America

## Abstract

FAK and Src signaling play important roles in cell differentiation, survival and migration. However, it remains unclear how FAK and Src activities are regulated at the initial stage of stem cell differentiation. We utilized fluorescence resonance energy transfer (FRET)-based FAK and Src biosensors to visualize these kinase activities at the plasma membrane of human mesenchymal stem cells (HMSCs) under the stimulation of osteogenic, myoblastic, or neural induction reagents. Our results indicate that the membrane FAK and Src activities are distinctively regulated by these differentiation induction reagents. FAK and Src activities were both up-regulated with positive feedback upon osteogenic induction, while myoblastic induction only activated Src, but not FAK. Neural induction, however, transiently activated FAK and subsequently Src, which triggered a negative feedback to partially inhibit FAK activity. These results unravel distinct regulation mechanisms of FAK and Src activities during HMSC fate decision, which should advance our understanding of stem cell differentiation in tissue engineering.

## Introduction

Human mesenchymal stem cells (HMSCs) can be renewed and allowed to differentiate into cells of mesenchymal as well as non-mesenchymal lineages, with ample applications in tissue engineering and regenerative medicine [1], [2]. The differentiation signals are transduced from the membrane receptors of HMSCs to transcription factors and genes in the nucleus, modulated by complex intracellular signaling networks [3], [4]. The importance of these regulatory signaling pathways in the development and maintenance of the skeleton, muscle, and neuron is widely accepted [5]. However, the mechanisms involved in HMSC differentiation and fate decision are just starting to emerge [6]–[8]. Therefore, monitoring and understanding the molecular events triggered at the beginning of HMSC differentiation will provide important insights for tissue engineering, regenerative medicine, and corresponding clinical applications.

The signaling pathways regulating stem cell differentiation can be initiated by cytokines, growth factors, or extracellular mechanical environment [7]–[9]. For example, FAK is a signaling mechano-sensing protein at the cell-matrix adhesion sites, which is known to form a complex with Src kinase and function at the focal adhesion sites to promote cell differentiation, migration, and proliferation [10]–[15]. Upon growth factor stimulation or cell-matrix interaction, the FAK/Src complexes can activate MAPK, which induces the phosphorylation of the transcription factor Runx2 and the subsequent osteogenesis process [16], [17]. On the other hand, Src is also an important signaling protein for maintaining bone homeostasis [5], [18], [19]. FAK and Src kinase activities have been found to be regulated independently during long-term osteogenetic differentiation [20]. Therefore, FAK and Src can regulate a cell's fate decision either cooperatively or independently.

However, many details at the initiation of the differentiation process, especially the crosstalk among these signaling pathways leading to transcriptional responses, remain unclear. For instance, the mechanisms how the differentiation induction reagents co-regulate the non-receptor protein kinases, including focal adhesion kinase (FAK) and Src, and how those signals initiate stem cell differentiation have not been fully understood. The roles played by FAK and Src during the myoblastic or neuronal differentiation remain largely unclear. In embryonic stem cells (ESCs), adhesion signals through FAK/Src are believed to negatively regulate their differentiation to cardiomyocytes [14], [21], [22], although a significant increase of FAK phosphorylation has been reported in maturing C_2_C_12_ myoblasts [23]. Meanwhile, FAK is considered a key protein during neurite differentiation and outgrowth in HMSCs [24], [25]. The inhibition of FAK-induced phosphorylation and FAK expression has been shown to prevent the neurite outgrowth in HMSCs induced by 2D and 3D matrix, respectively. Src tyrosine kinase was found to be involved in the neuronal differentiation of ESCs and PC12 cells, but has not been studied in HMSCs [26], [27]. Therefore, systematic investigation of FAK and Src activity in HMSCs with live cell imaging can provide crucial information to the tyrosine kinase signals at the initiation of differentiation.

Fluorescence resonant energy transfer (FRET)-based biosensors have been widely used to visualize molecular activities in live cells with high spatiotemporal resolution [28]–[30]. The FAK and Src FRET biosensors have been previously developed and extensively characterized by our group [11], [31], [32]. These biosensors utilize similar design strategies, both containing an enhanced cyan fluorescent protein (ECFP as the FRET donor), a Src SH2 domain, a flexible linker, a specific tyrosine-containing substrate peptide, and a variant of the yellow fluorescent protein (YPet as the FRET acceptor, Fig. S1A) [11], [20], [32]. Active FAK or Src can promote tyrosine phosphorylation on the substrate peptide of the corresponding biosensor, leading to a subsequent conformational change, and a decrease of FRET efficiency between the donor and the acceptor (Fig. S1B). Therefore, the donor/acceptor emission ratio can be used to represent Src or FAK activity in live cells [11], [31], [32]. Furthermore, these biosensors can be genetically engineered to localize to cytosol, plasma membrane, or organelles, providing versatile measurement of subcellular molecular activities. Therefore, FRET biosensors can provide powerful tools in deciphering the molecular mechanisms which initiate the differentiation processes.

Src biosensors have been applied to monitor force-stimulated Src activity in fibroblasts, and Src activity in HMSCs during long-term osteogenic differentiation [20], [33]. The membrane-tethered FAK and Src biosensors have been applied to monitor differentially regulated FAK/Src activation mechanisms with strong signals at the plasma membrane of live fibroblasts (Fig. S1A) [11], [34]. The sensitivity and specificity of the FAK biosensor has been extensively characterized *in vitro* and in FAK knockout cells re-constituted with various FAK mutants, to show that the observed increase in FRET ratio was due to the phosphorylation events caused by active FAK kinase [11]. Similarly, the sensitivity and specificity of the Src biosensor substrate sequence has also been extensively characterized *in vitro* and in mammalian cells [31]. In the current study, we utilized the membrane tethered Lyn-FAK and KRas-Src biosensors to visualize the FAK/Src activities and their inter-regulation at the plasma membrane of HMSCs under the stimulation of osteogenic, myoblastic, or neural induction reagents. Our results indicate that FAK and Src are distinctively regulated at the membrane of HMSCs treated with these differentiation reagents, which can provide crucial evidence on the early signals that lead to cell-fate decision at the initial stage of HMSC differentiation.

## Results

### The change of FAK and Src activities in HMSCs treated with osteogenic induction reagent

**Figure 1:**
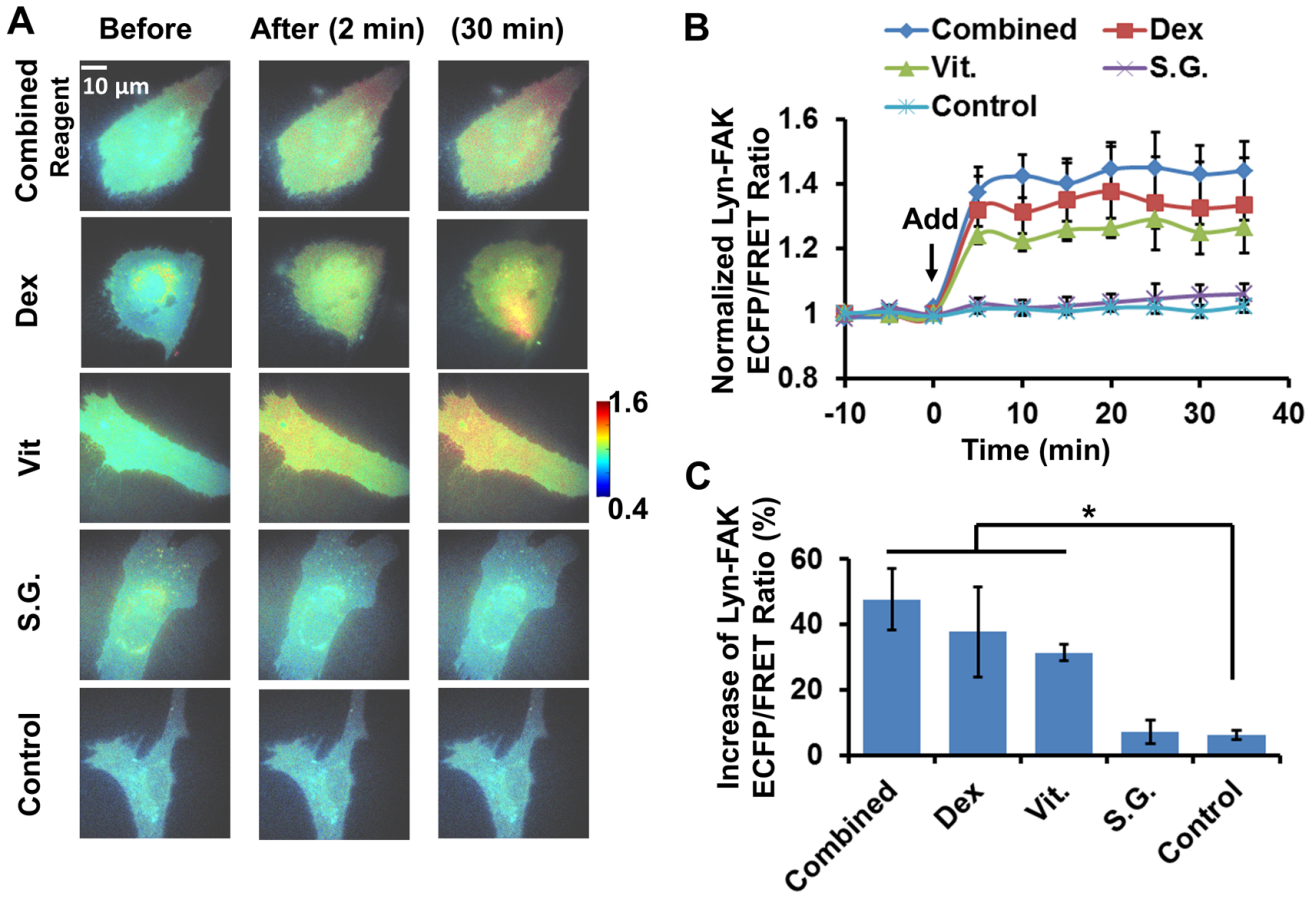
The effect of combined and independent components of osteogenic reagent on the Lyn-FAK activity in HMSCs. (**A**) The ECFP/FRET ratio images of Lyn-FAK biosensors in representative HMSCs before and after treatment. (**B**) The time courses of normalized Lyn-FAK ECFP/FRET ratio (mean ± SEM) in different experiments shown in panel (A). (**C**) The increase of ECFP/FRET ratio after treatment (mean ± SEM). (*) indicates significant difference between groups with p-value<0.05 by t-test.

To study FAK and Src activities during induced differentiation, we treated single live HMSCs with different components of the differentiation induction reagents. First, the effects of the combined osteogenic mixture and its components on FAK activity were examined in HMSCs expressing the Lyn-FAK biosensor. The combined osteogenic induction mixture contains three chemical components: Dexamethasone (DEX), Vitamine C (Vit) and β-sodium glycerophosphate (SG, a classical serine-threonine phosphatase inhibitor and organic phosphate donor) [35]–[37]. As shown in Figure 1, the combined osteogenic mixture and its components, DEX and Vit, but not the SG component, induced a significant increase of ECFP/FRET ratio in cells with FAK biosensors, indicating an increase of FAK activity at the cells membrane. The treatment of combined reagent, DEX, or Vit, induced in 10 minutes an average 30–50% increase of the FAK ECFP/FRET ratio in the cells. This response remained at a steady level for about 30–40 minutes during imaging (Fig. 1B). The responses of FAK ECFP/FRET ratio to these three treatments were not significantly different from each other (Fig. 1C). These results indicate that FAK in the HMSCs was significantly activated by the combined osteogenic mixture, probably attributing to its individual components DEX and Vit, but not SG.

**Figure 2:**
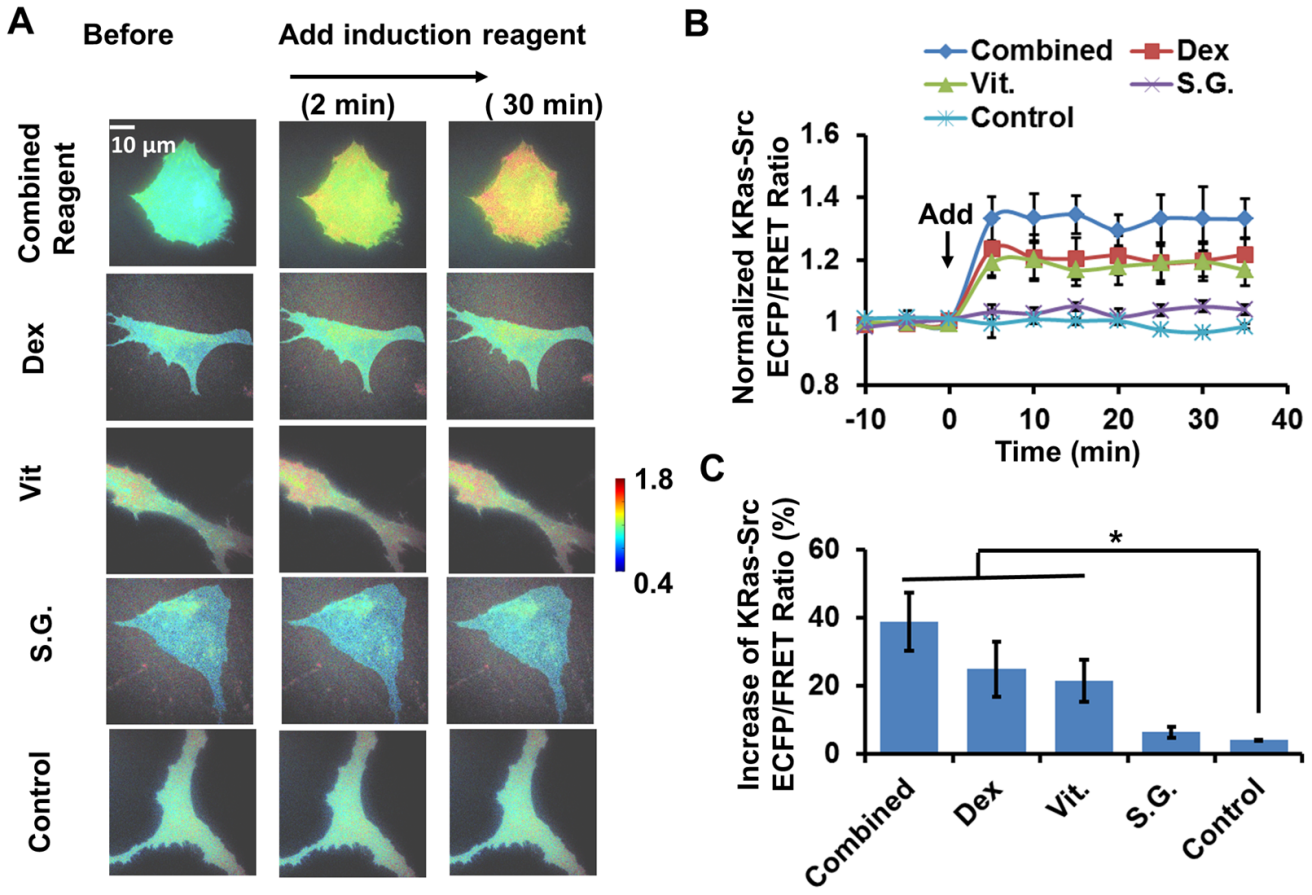
The effect of combined and independent components of osteogenic reagent on KRas-Src activity in HMSCs. (**A**) The ECFP/FRET ratio images of KRas-Src biosensors in representative HMSCs before and after treatment. (**B**) The time courses of normalized KRas-Src ECFP/FRET ratio (mean ± SEM) in different experiments shown in panel (A). (**C**) The increase of ECFP/FRET ratio after treatment (mean ± SEM). (*) indicates significant difference between groups with p-value<0.05 by t-test.

The osteogenic induction reagent had a similar effect on the membrane Src activity in HMSCs. As shown in Figure 2, the Src ECFP/FRET ratio significantly increased after the treatment of the combined osteogenic mixture, or its individual components DEX or Vit except SG. All three kinds of treatment induced an average 20–40% increase of the Src ECFP/FRET ratio in single HMSCs, which remained at a steady level for 30–40 minutes during imaging (Fig. 2B). Again, the effects of the three treatments on the HMSC Src ECFP/FRET ratio were not significantly different from each other (Fig. 2C). These results indicate that membrane Src activity in the HMSCs were significantly up-regulated by the combined osteogenic mixture, probably attributing to DEX and Vit, but not SG.

**Figure 3:**
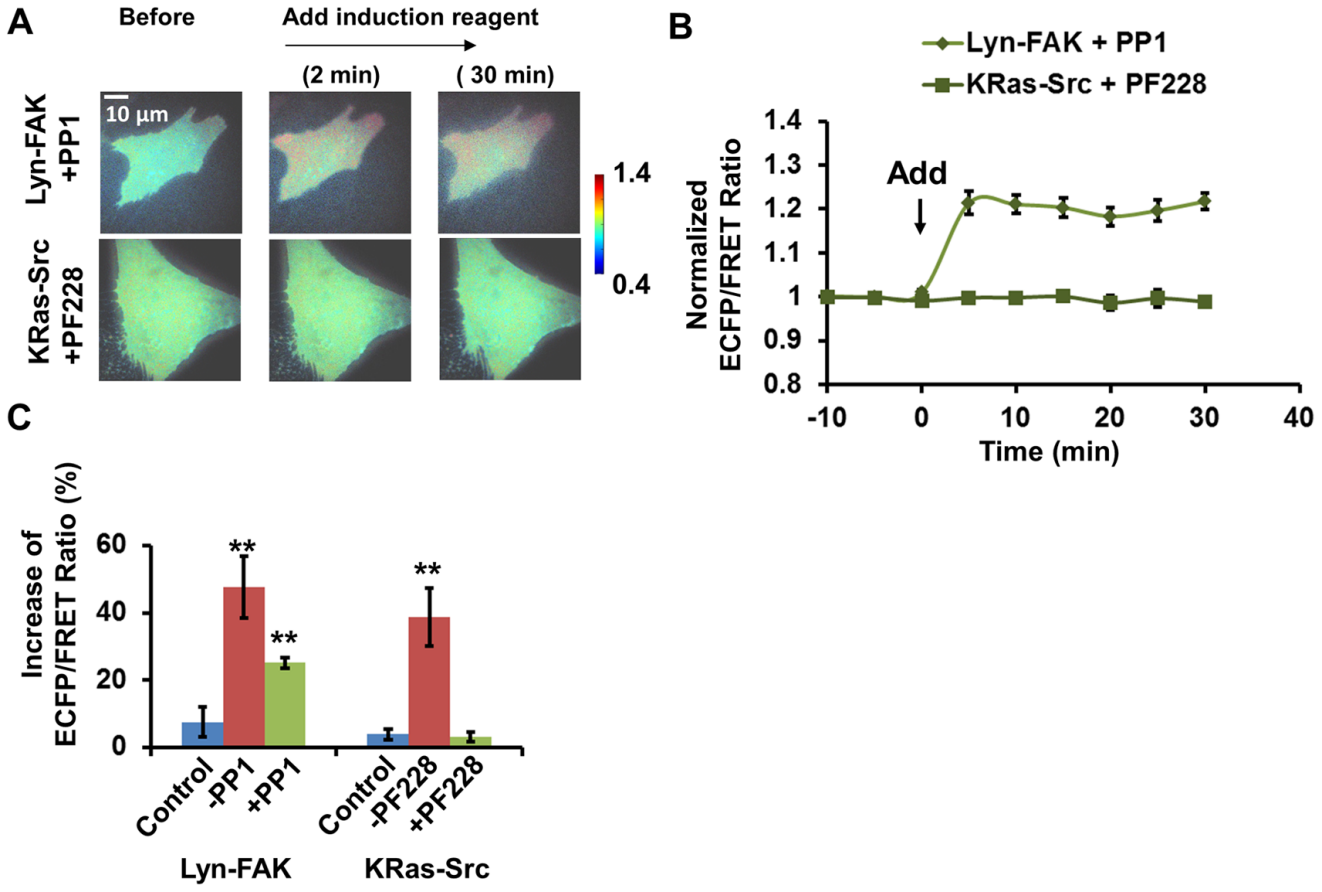
FAK/Src inter-regulation in HMSCs treated with combined osteogenic reagent. (**A**) The emission ratio images of the Src and FAK biosensors in HMSCs cross-inhibited by FAK and Src inhibitors with treatment of combined osteogenic reagent, respectively. (**B**) The time courses of the normalized emission ratio of the Src/FAK biosensor (mean ± SEM) in HMSCs shown in panel (A). (**C**) The change of the Src/FAK ECFP/FRET ratio (mean ± SEM) in HMSCs shown in panel (A). (**) indicates a group is significantly different from other groups in the same cluster, p-value<0.05 by t-test.

To understand the signaling pathway between FAK and Src at the initial status of HMSCs differentiation, we cross-inhibited FAK or Src activity in the cells under treatment. Briefly, HMSCs with Lyn-FAK biosensor were pre-treated by PP1 (10 μM) [31], a Src inhibitor, to examine whether Src activation was an upstream signal of FAK activation during osteogenic induction. Similarly, the HMSCs with KRas-Src biosensor were pre-treated with a FAK inhibitor PF228 (1 μM) [11] to examine the importance of FAK activity in Src activation. In HMSCs pretreated with PF228, the increase of Src ECFP/FRET ratio was completely inhibited, indicating the FAK activity is an upstream signal necessary for Src activation. In contrast, in cells pretreated with PP1, the increase of FAK ECFP/FRET ratio was only partially inhibited. FAK ECFP/FRET ratio increased ∼20% after the addition of the combined osteogenic mixture in these cells, which is significantly less than that in cells without PP1 treatment (Fig. 3C). This result indicates that the FAK activation by the combined mixture was partially dependent on the feedback from Src activity, since FAK activation was reduced when PP1 was present (Figs. 3B and 3C). Nevertheless, there was also a portion of FAK activity that was independent of Src, since the FAK activation was still seen with the presence of PP1 (Figs. 3B and 3C).

Taken together, these results suggest that osteogenic induction reagent significantly activated both FAK and Src kinases at the membrane of HMSCs. Activated FAK kinase can up-regulate Src kinase activity, which then positively feedbacks and causes further activation of FAK in the cells. This result also supports a previous report that bone induction reagent increased FAK phosphorylation [38].

### The change of FAK and Src activities in HMSCs treated with myoblast induction reagent

**Figure 4:**
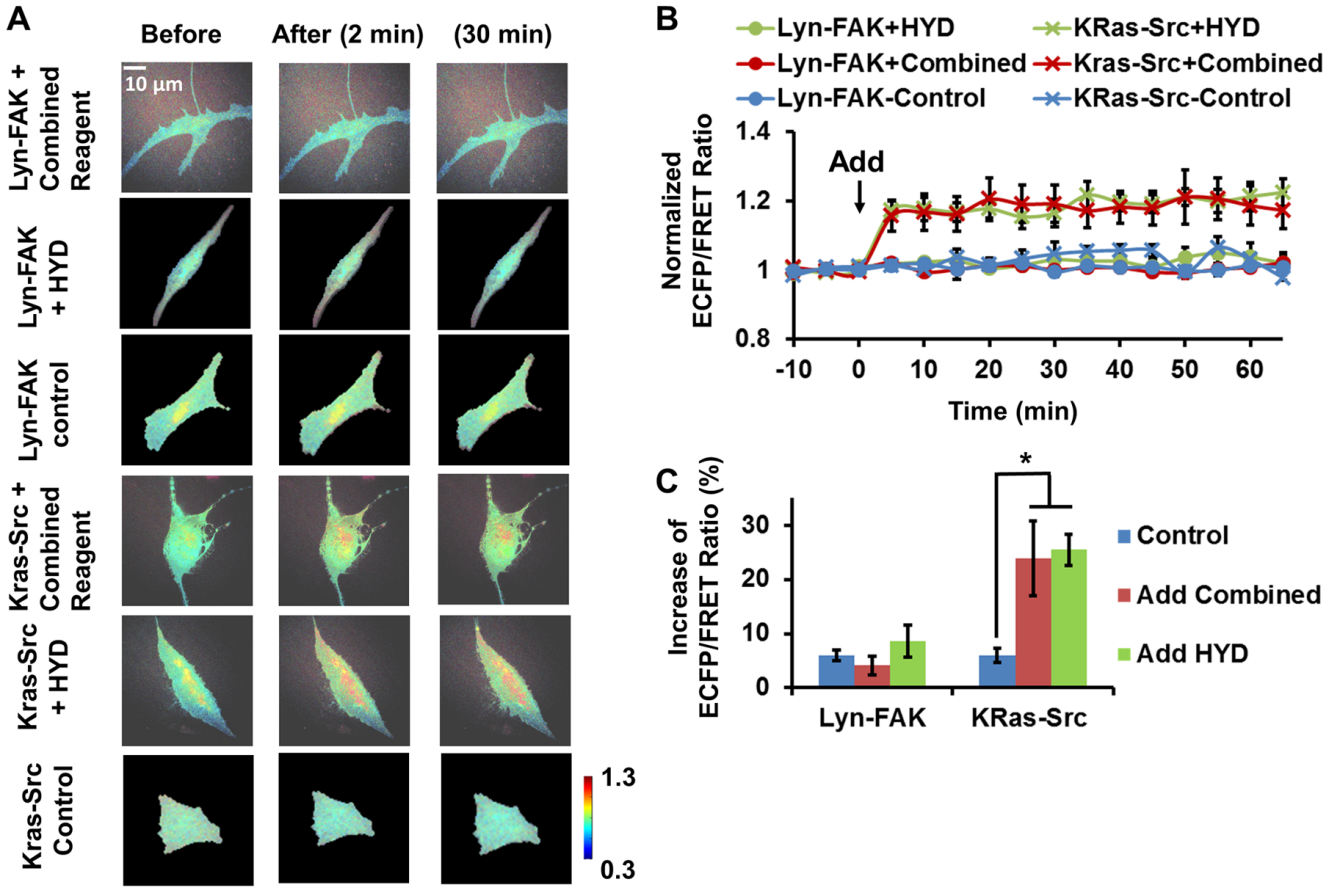
The effect of combined and independent components of muscle induction reagent on FAK and Src activity in HMSCs. (A) The ECFP/FRET ratio images of the Src/FAK biosensor in representative HMSCs before and after treatment. (B) The time courses of normalized Src/FAK ECFP/FRET ratio (mean ± SEM) in different experiments shown in panel (A). (C) The increase of Src/FAK ECFP/FRET ratio after treatment (mean ± SEM). (*) indicates significant difference between groups with p-value<0.05 by t-test.

The combined myoblast induction mixture consists of two components, Hydrocortisone (HYD) and DEX [9]. The combined mixture and the individual components were each used to stimulate HMSCs to visualize the induced change of FAK/Src activity. Compared with osteogenic induction, the response of FAK and Src activities are different toward myoblastic induction. As shown in Figure 4, the FAK ECFP/FRET ratio did not change significantly with the stimulation of the combined mixture or HYD, indicating that FAK activity in HMSCs was not affected by these treatments. Since DEX is also a component of the osteogenic reagent, its effect was examined in the experiments of osteogenic induction reagents (Figs. 1 and 2). The results show that DEX induced ∼35% increase of FAK activity, but this increase was blocked by HYD in the combined mixture. In contrast, the Src ECFP/FRET ratio increased by ∼25% on average to a steady level in HMSCs treated with either the combined mixture or HYD, as well as DEX, indicating that Src was significantly activated by the muscle induction reagents, possibly attributing to both HYD and DEX (Figs. 3 and 4).

**Figure 5:**
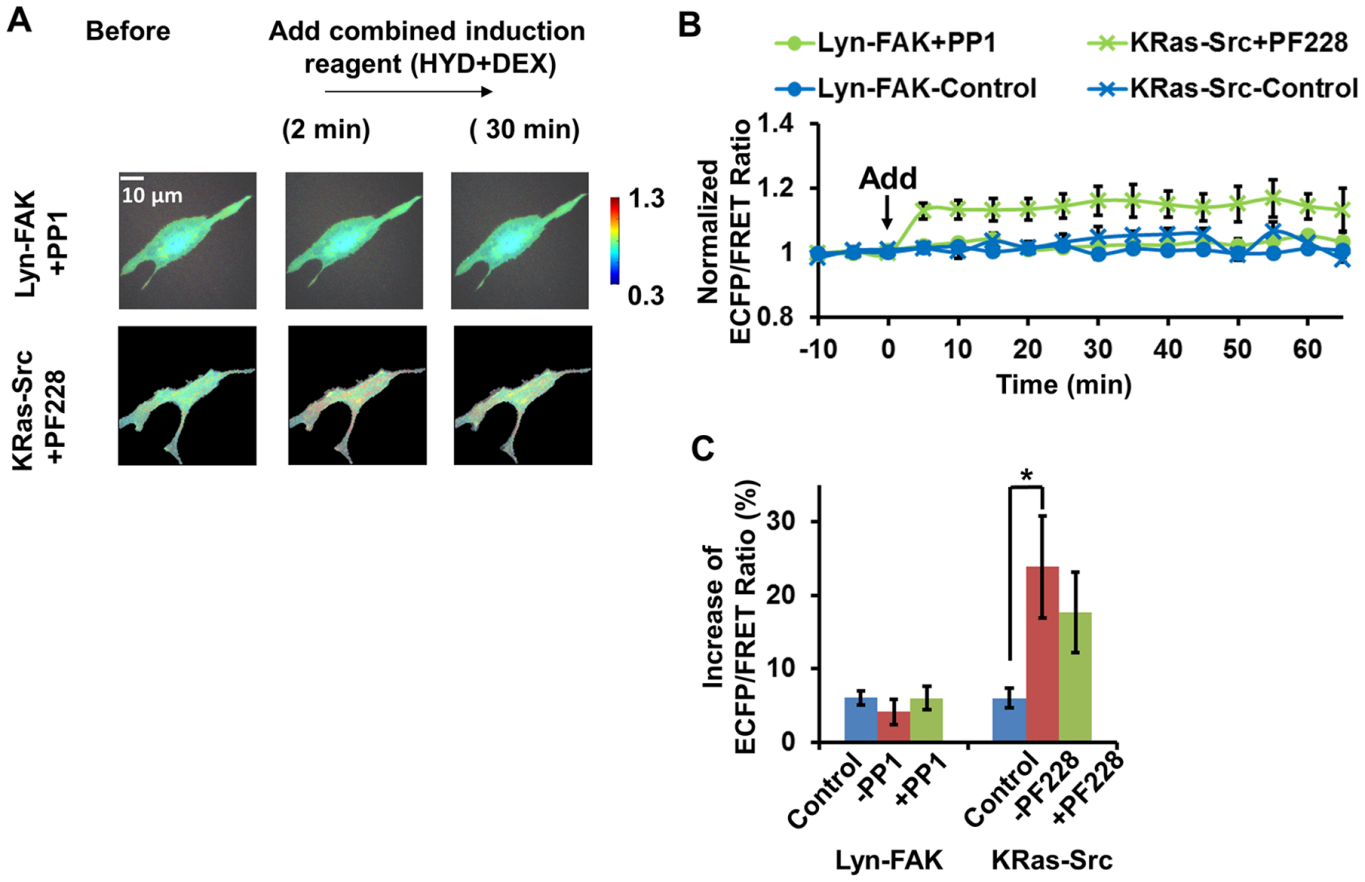
FAK/Src inter-regulation in HMSCs treated with combined muscle induction reagent. (**A**) The emission ratio images of the Src and FAK biosensors in HMSCs cross-inhibited by FAK- and Src inhibitors respectively with treatment of combined muscle induction reagent. (**B**) The time courses of the normalized emission ratio of the Src/FAK biosensor (mean ± SEM) in HMSCs shown in panel (A). (**C**) The change of the Src/FAK ECFP/FRET ratio (mean ± SEM) in HMSCs shown in panel (A). (*) indicates significant difference between groups with p-value<0.05 by t-test.

Similar to the experiments of osteogenic induction, we performed the cross-inhibition experiments to study Src and FAK signaling. As expected, the increase of Src ECFP/FRET ratio induced by the combined mixture was not significantly inhibited by the FAK inhibitor PF228, indicating that the induced Src activation was relatively independent of FAK kinase activity at the cellular membrane (Fig. 5). Therefore, we conclude that the combined myoblastic induction mixture activated Src kinase independent of FAK, while they did not activate FAK in the HMSCs.

### The change of FAK and Src activities in HMSCs treated with neuron induction reagent

**Figure 6:**
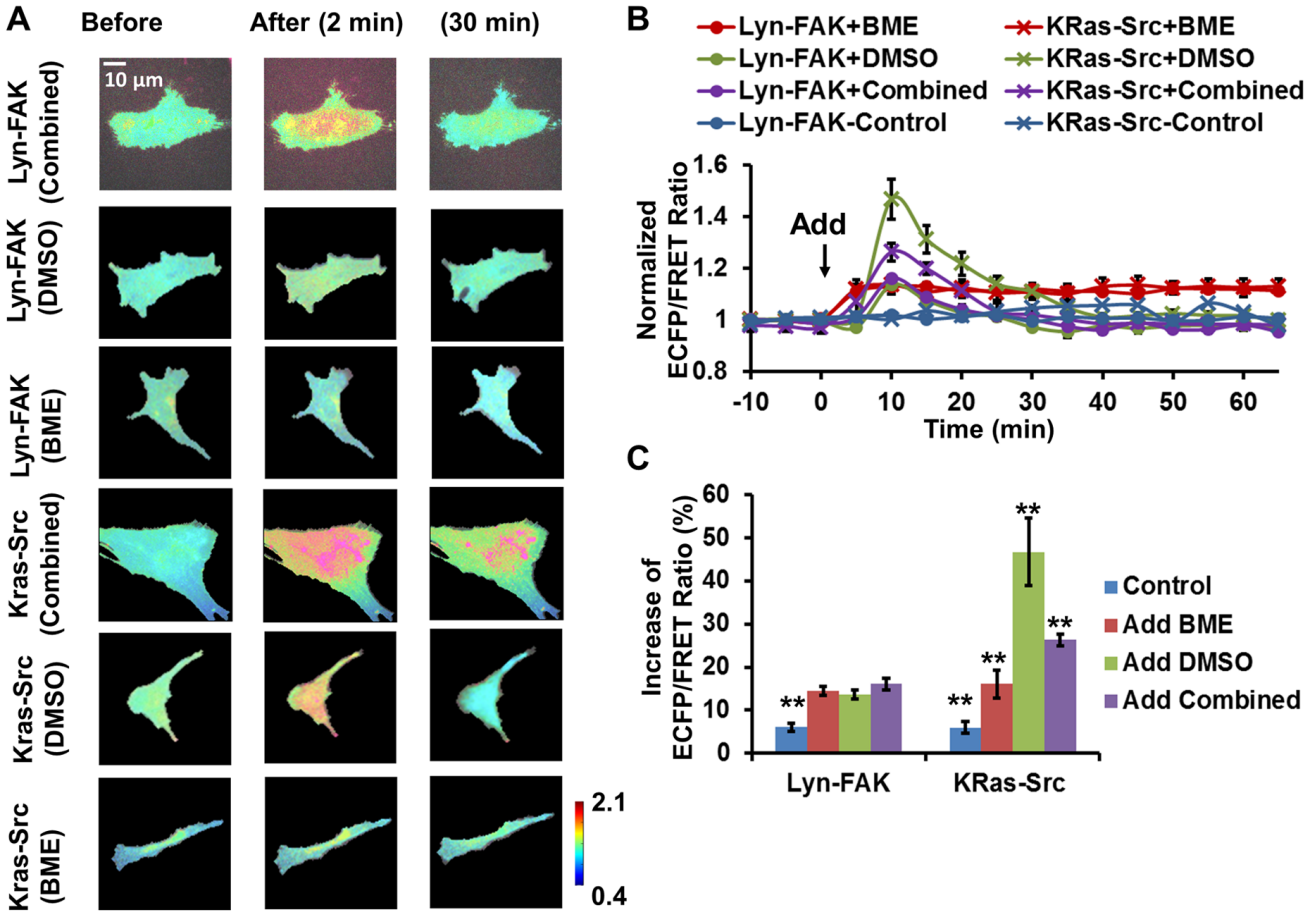
The effect of combined and independent components of neural induction reagent on FAK and Src activity in HMSCs. (A) The ECFP/FRET ratio images of the Src/FAK biosensor in representative HMSCs before and after treatment. (B) The time courses of normalized Src/FAK ECFP/FRET ratio (mean ± SEM) in different experiments shown in panel (A). (C) The increase of Src/FAK ECFP/FRET ratio after treatment (mean ± SEM). (*) indicates significant difference between groups with p-value<0.05 by t-test; (**) indicates a group is significantly different from other groups in the same cluster, p-value<0.05 by t-test.

MSCs can also be induced to differentiate into neuronal cells with the addition of neural induction reagents containing 2-mercaptoethanol (BME) and dimethyl sulfoxide (DMSO) [39], [40]. The combined neural induction mixture and its individual components DMSO and BME were each used to treat the HMSCs. Surprisingly, both the combined mixture and DMSO induced a significant (∼15%) yet *transient* increase of the FAK ECFP/FRET ratio in the HMSCs, which peaked around 10 minutes and largely declined to the basal level around 35 minutes after stimulation. BME, on the other hand, induced a ∼15% increase of the FAK ECFP/FRET ratio, which remained *steady* during imaging (Fig. 6). These results indicate that the combined neural induction mixture activated FAK at the membrane of HMSCs with a *transient* kinetics, possibly due to the dominant effect of DMSO. As for Src kinase, the combined mixture also induced a significant (∼25%) and transient increase of the Src ECFP/FRET ratio in HMSCs, while the transient peak of ratio induced by DMSO was significantly higher (∼45%). BME also induced an increase of the Src ECFP/FRET ratio and the increase remained stable at ∼15%. Therefore, these results suggest that in the combined mixture, BME damped the effect of DMSO, and that Src kinase activity was transiently enhanced by neural induction at the membrane of HMSCs.

**Figure 7:**
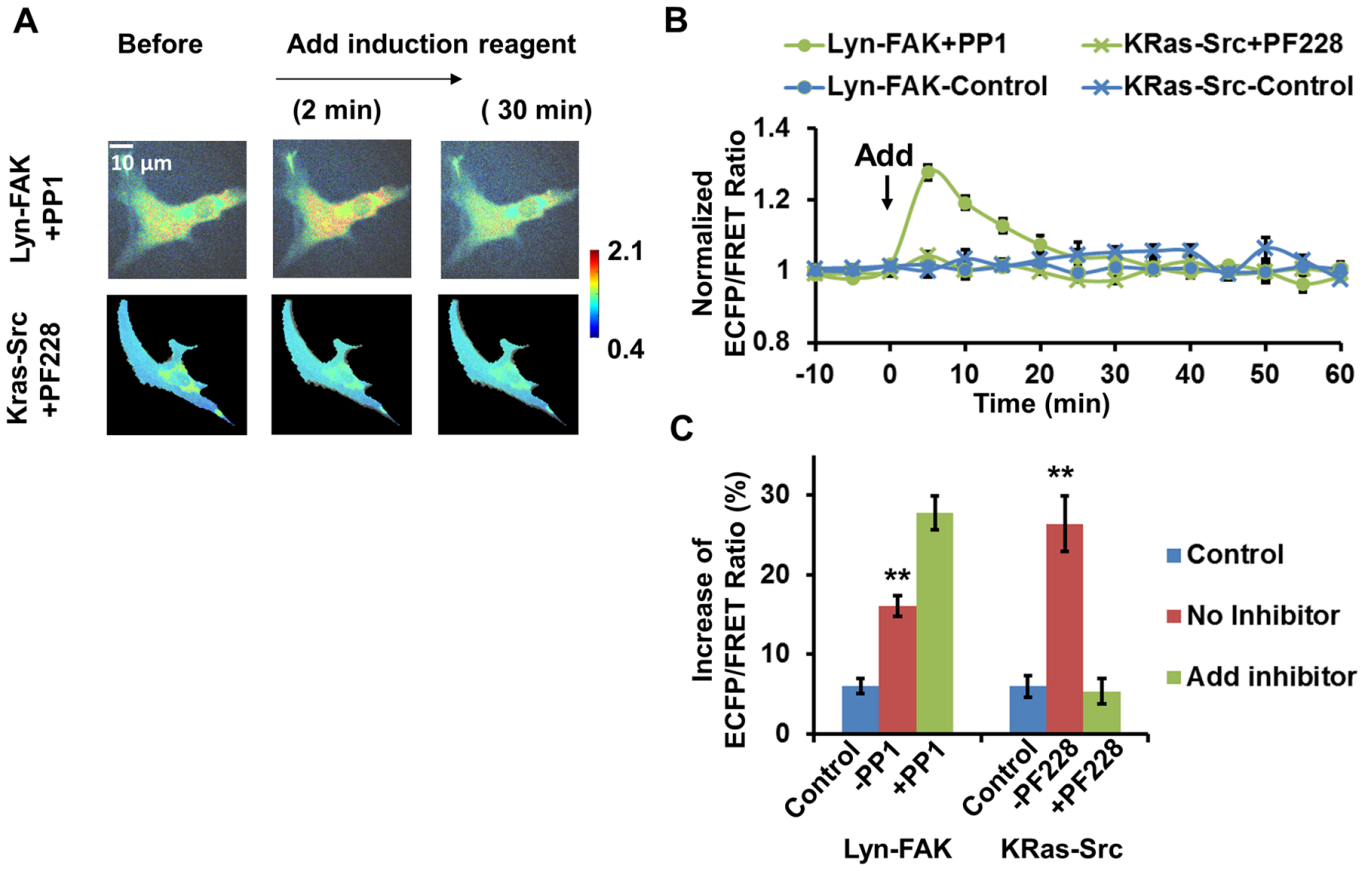
FAK/Src inter-regulation in HMSCs treated with combined neural induction reagent. (**A**) The emission ratio images of the Src and FAK biosensors in HMSCs cross-inhibited by FAK and Src inhibitors respectively with treatment of combined neural induction reagent. (**B**) The time courses of the normalized emission ratio of the Src/FAK biosensor (mean ± SEM) in HMSCs shown in panel (A). (**C**) The change of the Src/FAK ECFP/FRET ratio (mean ± SEM) in HMSCs shown in panel (A). (**) indicates a group is significantly different from other groups in the same cluster, p-value<0.05 by t-test.

Again the cross-inhibition experiments were performed to probe the FAK/Src interaction during neural induction. Intriguingly, in HMSCs treated with the Src inhibitor PP1, the FAK ECFP/FRET ratio increased to a significantly higher level (25%) after neural induction than non-inhibited HMSCs (15%, Fig. 7), suggesting that FAK was hyperactive in cells without Src activity. Furthermore, when the HMSCs were treated with FAK inhibitor, the induced increase of the Src ECFP/FRET ratio was completed blocked (Fig. 7). This suggested that FAK activity was necessary for Src activation in the HMSCs during neural induction. Taken together, these results indicate that the neural induction reagents induced FAK kinase activity, which can up-regulate downstream Src kinase activation. The negative feedback from Src kinase activity partially reduced FAK activation, which may contribute to the eventual stabilization of the cellular system and bring the activated kinases down to the basal level.

**Figure 8:**
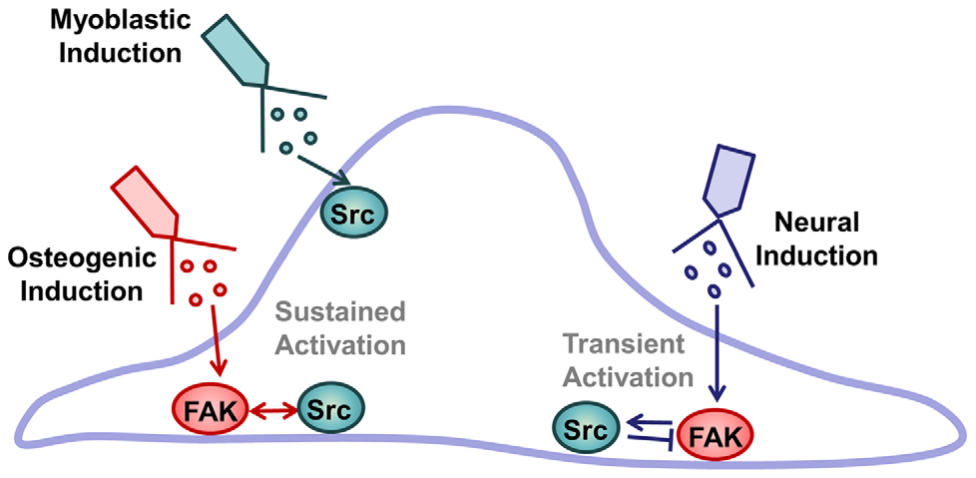
FAK/Src activation mechanism upon differentiation induction. The schematics diagram showing different activation patterns of FAK/Src with distinct regulation mechanisms upon the treatment of different induction medium on HMSCs.

In summary, we used FRET-based biosensors to report the FAK/Src kinase activity in HMSCs treated by different differentiation induction medium. The results indicate different activation pattern of FAK/Src with distinct regulation mechanisms. Upon osteogenic induction, FAK kinase was activated upstream of Src kinase, with Src activity feedback and further activating FAK in HMSCs (Fig. 8). In contrast, during myoblastic induction, Src kinase was activated independently while FAK remains inactive (Fig. 8). During neural induction, FAK was activated upstream of Src, while Src kinase activity negatively feedback and damped FAK activity (Fig. 8).

## Discussion

At the current stage, tissue engineering and stem-cell-based therapies involve multidisciplinary areas, including material science, bioengineering, biology and medicine [2], [4]. Prominent clinical applications include the successful cellularization of donor trachea with MSC-derived cartilage-like cells [41], and the improvement of ventricular function by injected cardiac stem cells [42]. However, it remains a challenge to understand the interaction between matrix property and differentiation induction factor. For example, why did the MSCs differentiate into bone-like cells, but not cardiac cells, at the site of scar tissue generated by cardiac infarction [43]? Therefore, understanding the response of MSCs to chemical-induced differentiation, especially the sub-cellular and molecular signaling pathways, may advance our understanding on the stem cell fate decision and differentiation in clinical applications.

It has been reported that bone induction media containing dexamethasone can promote FAK tyrosine phosphorylation [38], [44]. In addition, FAK can be activated during the osteogenesis process induced by shear stress or stiff extracellular matrix [9], [10], [45]. We previously showed that FAK was continuously activated during long-term differentiation induced by osteogenic reagents, but the effect of myoblastic or neural differentiation medium on FAK kinase activity was not clear. Our results showed that FAK activity was up-regulated up to 140% of the basal level by the components of osteogenic reagents, but not the myoblastic induction reagents. In contrast to the strong and stable up-regulation of osteogenic reagents, components of neurogenic reagents caused a sometimes transient up-regulation of FAK activity up to 120% of the basal level. It is possible that stable FAK activation can promote the mechano-sensitivity of the cells in bone tissue, but not preferred during myogenic or neural differentiation [46].

Src is a non-receptor protein tyrosine kinase which plays important roles in cell migration and differentiation [5], [15], [47]. Its long-term expression and activity have been found to inhibit the differentiation of HMSCs toward osteoblasts [18], [19]. However, it remains unclear how Src activity is regulated at the initiation of reagent-induced differentiation. We previously showed that Src kinase activity was continuously down-regulated in HMSC cytosol during long-term osteogenic differentiation, independently of the FAK activation [20]. Here, our results suggest that Src activity at cellular membrane is up-regulated in concert with FAK at the initiation of osteogenic induction, but independent of FAK at the initiation of myoblastic induction. It is possible that Src activity at plasma membrane can be different from that in the cytosol, while the subcellular coordination of Src activity between 1 hour and 8 days after the osteogenic induction remains unclear. Upon neural induction, intriguingly, Src activity was only up-regulated transiently with a negative feedback to its upstream FAK activity, which probably contributes to damping the FAK/Src signals following the transient activation.

It is possible that FAK and Src can cooperate and promote the downstream phosphorylation events which lead to the fate decision during stem cell osteogenesis. For example, active FAK can facilitate auto-phosphorylation at its tyrosine site Y397, which recruits and activates Src kinase via Src SH2 domain, with active Src further promotes the phosphorylation of FAK at the tyrosine sites Y576 and Y577 and the subsequent maximal activation of FAK kinase [11], [48]. Down-stream of FAK/Src activity, β-catenin phosphorylation and stabilization can promote osteogenic differentiation while suppressing adipogenic and neuronal differentiation [8], [49]. Active Src can promote β-catenin expression and phosphorylation in cancer cells [50], [51]. FAK/Src signaling is required for β-catenin-induced mobilization and proper differentiation [52]. Although FAK and Src have been shown to cooperate during osteogenic differentiation at focal adhesion sites, our results clearly indicate that their activities are distinctively regulated during the induced myoblastic and neural differentiation of HMSC. This is also consistent with recent findings that Src and FAK reside at different membrane microdomains and that certain Src function is not localized at focal adhesion sites as FAK [11], [34], [53]. It is hence possible that only a fraction of Src and FAK co-localize and function in coordination in cells under certain conditions, e.g. at the initiation of osteogenic induction. Intriguingly, Src activation provided negative feedback to FAK activation during neuronal induction. It is possible that the phosphorylation events downstream of Src kinase can disrupt focal adhesion complex which subsequently down-regulates FAK activity [54], [55]. These different modules of FAK and Src signaling complex may result in their distinct activation pattern and regulation mechanisms at the initiation of induced myoblastic and neural differentiation of HMSCs.

In summary, our results showed that FAK and Src activities were differentially regulated in HMSCs stimulated by different differentiation-induction reagents. These distinct inter-regulation patterns between FAK and Src activation suggest novel and sometimes un-coupled Src/FAK functions during HMSC differentiation process. This result of the regulation of FAK and Src activities is also consistent with the roles played by the FAK/Src complex in stabilizing Wnt/β-catenin signaling, the subsequent transcription regulation, and the fate decision of the stem cells. Further study on the signaling pathways connecting FAK/Src to gene regulation during HMSCs differentiation can greatly benefit the area of tissue engineering and stem-cell based therapy.

## Materials and Methods

### Cell culture, induction reagent and inhibitors

The HMSCs were purchased from ATCC. The cell culture reagents were obtained from Invitrogen. Cells were maintained in DMEM supplemented with 10% fetal bovine serum (FBS), sodium pyruvate (1 mM), penicillin (1 U/ml), and L-glutamine (2 mM). Cells were cultured in a humidified 5% CO_2_ (and 95% air) incubator at 37°C. The differentiation of HMSCs was induced with the combined mixture: (1) for osteoblastic induction: dexamethasone (DEX, 10^−7^ M), ascorbic acid (Vit, 50 mg/L, a form of vitamin C), β-sodium glycerophosphate (SG, 10 mM) and ascorbate 2-phosphate) [35]–[37]; (2) for myoblastic induction: dexamethasone (DEX, 10^−7^ M), Hydrocortisone (HYD, 50 µM) [9]; (3) for neural induction: 2-mercaptoethanol (BME, 6 mM), dimethyl sulfoxide (DMSO, 2%), and beta-hydroxyanisol (BHA 200 uM, an antioxidant and protective reagent) [39], [40]. A specific Src inhibitor PP1 (10 μM, BioMol) or a specific FAK inhibitor PF228 (1 μM) was used to pretreat the cells for 1 hour before imaging [56], [57]. Both the FAK and Src specific inhibitors have been used in our published work where they were fully functional at the above concentrations [11], [31].

### Gene construction and DNA plasmids

The FAK and Src biosensors were previously developed and characterized [11], [32]. As shown in Figure S1, Src and FAK biosensors were constructed by fusing the SH2 domain from c-Src, a flexible linker, and a specific substrate peptide between the N-terminus ECFP and the C-terminus YPet [11], [20], [31], [32], [34]. The DNA encoding the Src/FAK biosensors were sub-cloned with the BamH1/EcoR1sites in pRSetB for the protein purification from *E-coli*, and in pcDNA3 plasmid for the expression and localization in the cytosol of mammalian cells [11], [32].

### Microscopy image acquisition and quantification

The HMSCs were cultured in DMEM supplemented with 10% FBS (GIBCO) at 37°C. The cells were transfected with the Lyn-FAK or KRas-Src biosensor 36–48 hours before imaging and transferred into cover-glass-bottom dishes (Cell E&G) with starvation medium (DMEF with 0.5% FBS)12–24 hours after transfection. During imaging, the cells were kept in CO_2_-independent medium with 0.5% FBS. The ECFP and FRET fluorescence intensity images were acquired with a Nikon microscope, a charge-coupled device (CCD) camera, and the MetaFluor 6.2 software (Universal imaging). The fluorescence intensity images were then background subtracted and used to compute the pixel-wise ECFP/FRET ratio images. The time course of ECFP/FRET ratio in each cell was obtained in a randomly selected region of interest in the cell body and normalized such that the average ratio value before treatment is 1. The normalized time courses from different cells in the same group are then averaged and plotted in Excel (Microsoft). The maximal changes of ECFP/FRET time course were calculated for each cell and averaged to obtain the response of ECFP/FRET ratio shown in panel (C) in Figures 1, 2, 3, 4, 5, 6, 7. The statistical comparison of the maximal change of ECFP/FRET ratio was performed with one tailed t-tests.

## Supporting Information

Figure S1 The design principle of the FAK and Src biosensors. (**A**) The schematics of the FAK and Src biosensors shown with their specific substrate sequences; (**B**) The activation mechanism of the FAK and Src biosensors; (**C**) The schematic design of the Lyn-tagged and KRas-tagged biosensors and their membrane localization.
(TIF)
