# Supplementary figures and images for: The Effect of Differentiation Induction on FAK and Src Activity in Live HMSCs Visualized by FRET
Source: PLoS One. 2013 Aug 27;8(8):e72233. doi: 10.1371/journal.pone.0072233 (PMC3754985; doi:10.1371/journal.pone.0072233)

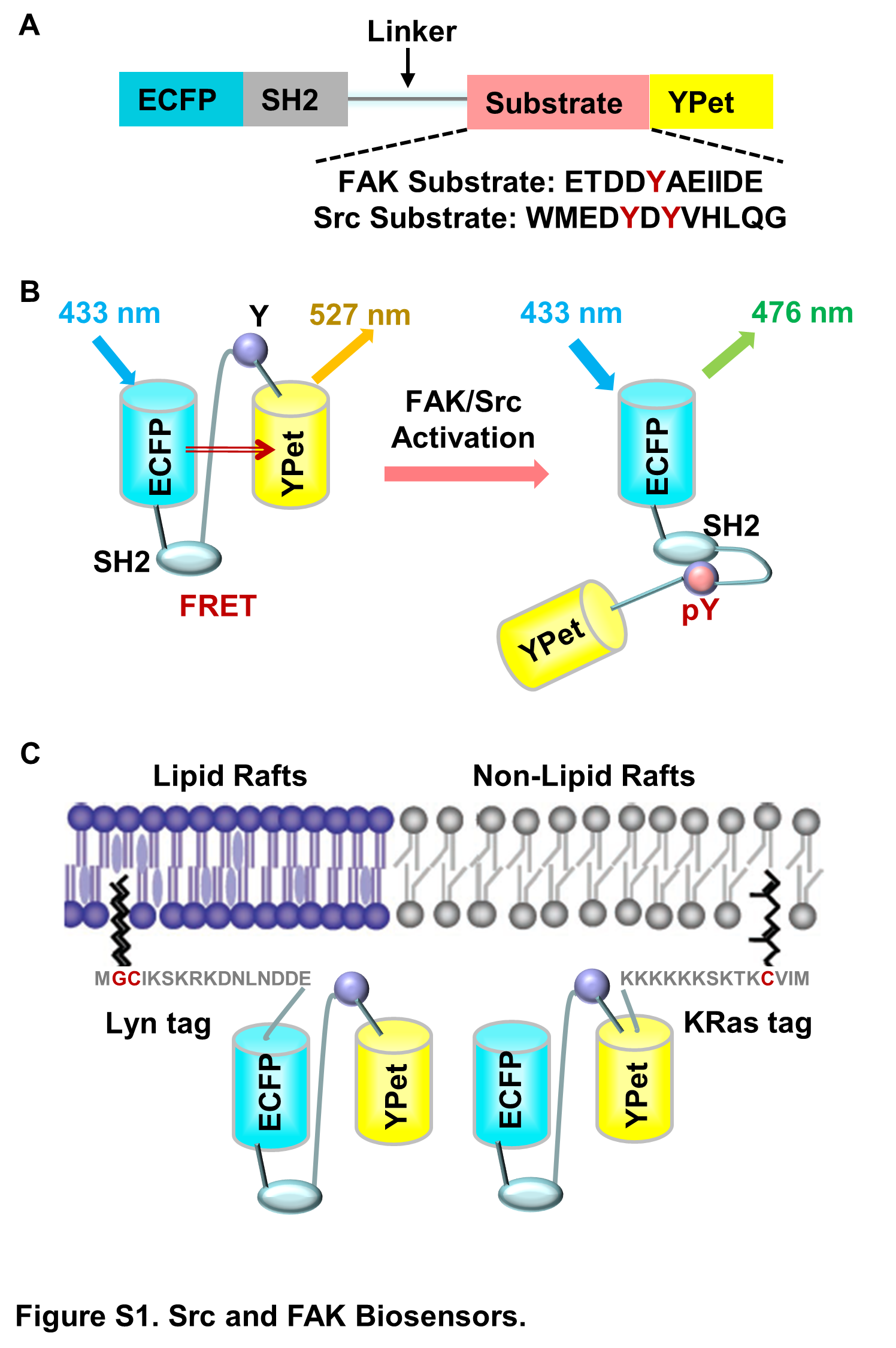

Supplement: Figure S1 — The design principle of the FAK and Src biosensors. (A) The schematics of the FAK and Src biosensors shown with their specific substrate sequences; (B) The activation mechanism of the FAK and Src biosensors; (C) The schematic design of the Lyn-tagged and KRas-tagged biosensors and their membrane localization. (TIF) [file pone.0072233.s001.tif]
